# NS5 Conservative Site Is Required for Zika Virus to Restrict the RIG-I Signaling

**DOI:** 10.3389/fimmu.2020.00051

**Published:** 2020-02-14

**Authors:** Aixin Li, Wenbiao Wang, Yingchong Wang, Keli Chen, Feng Xiao, Dingwen Hu, Lixia Hui, Weiyong Liu, Yuqian Feng, Geng Li, Qiuping Tan, Yingle Liu, Kailang Wu, Jianguo Wu

**Affiliations:** ^1^State Key Laboratory of Virology, College of Life Sciences, Wuhan University, Wuhan, China; ^2^Guangzhou Key Laboratory of Virology, Institute of Medical Microbiology, Jinan University, Guangzhou, China; ^3^Guangdong LongFan Biological Science and Technology Company, Foshan, China

**Keywords:** IRF3, IFN-β, methyltransferase, NS5, RIG-I, ubiquitination, ZIKV

## Abstract

During host–virus co-evolution, cells develop innate immune systems to inhibit virus invasion, while viruses employ strategies to suppress immune responses and maintain infection. Here, we reveal that Zika virus (ZIKV), a re-emerging arbovirus causing public concerns and devastating complications, restricts host immune responses through a distinct mechanism. ZIKV nonstructural protein 5 (NS5) interacts with the host retinoic acid-inducible gene I (RIG-I), an essential signaling molecule for defending pathogen infections. NS5 subsequently represses K63-linked polyubiquitination of RIG-I, attenuates the phosphorylation and nuclear translocation of interferon regulatory factor 3 (IRF3), and inhibits the expression and production of interferon-β (IFN-β), thereby restricting the RIG-I signaling pathway. Interestingly, we demonstrate that the methyltransferase (MTase) domain of NS5 is required for the repression of RIG-I ubiquitination, IRF3 activation, and IFN-β production. Detailed studies further reveal that the conservative active site D146 of NS5 is critical for the suppression of the RIG-I signaling. Therefore, we uncover an essential role of NS5 conservative site D146 in ZIKV-mediated repression of innate immune system, illustrate a distinct mechanism by which ZIKV evades host immune responses, and discover a potential target for anti-viral infection.

## Introduction

Zika virus (ZIKV), a re-emerging arbovirus, has raised public concerns due to its global spread and clinical symptoms. Since 2007, ZIKV infection has caused a series of epidemics in Micronesia, the South Pacific, and most recently the Americas (1). The viral infection is responsible for the development of devastating complications, including Guillain–Barre Syndrome (2), meningoencephalitis in adults, and microcephaly in fetuses (3), as well as testis damage and infertility in male mice (4). ZIKV is a single-stranded positive-sense RNA virus (5), and its genome contains a 5′-untranslated region (UTR), an open reading frame (ORF), and a 3′-UTR (6). The ORF encodes a single polyprotein that is processed into capsid protein (C), precursor membrane protein (prM), envelope protein (E), and nonstructural proteins (NS1, NS2A, NS2B, NS3, NS4A, NS4B, and NS5) (7).

As an early response to virus infection, the host immune system detects viral molecules through the pathogen-associated molecular patterns (PAMPs), which are recognized by the pattern recognition receptors (PRRs) (8). The well-known PRRs are the toll-like receptors (TLRs) (9), the retinoic acid-inducible gene I (RIG-I)-like receptors (RLRs) (10), the nucleotide-binding oligomerization domain (NOD)-like receptors (NLRs) (11), and the C-type lectin receptors (CLRs) (12). The RLRs comprising of RIG-I, melanoma differentiation-associated protein 5 (MDA5), and laboratory of genetics and physiology 2 (LGP2) detect viral RNA in most cell types (13). Upon sensing viral RNA, RIG-I dephosphorylation occurs, which triggers RIG-I polyubiquitination by two ubiquitin E3 ligases, tripartite-motif protein 25 (TRIM25), and Riplet (14). Polyubiquitinated RIG-I interacts with mitochondrial antiviral-signaling protein (MAVS) and activates the TANK-binding kinase 1 (TBK1) and the inhibitor of κB kinase ε (IKKε), leading to the phosphorylation of interferon regulatory factor 3 (IRF3) (15–17). Phosphorylated IRF3 is subsequently translocated into the nucleus and induces the production of type I interferons (IFNs), which binds to their receptors to induce interferon-stimulated genes (ISGs), resulting in antiviral responses (18, 19).

During infection, ZIKV RNA is recognized by RIG-I (20). ZIKV has developed several strategies to limit host immune responses and to successful replicate and spread (21). ZIKV NS5 suppresses type I IFN by targeting STAT2 for degradation (22). An antagonistic system employing multiple ZIKV NS proteins restricts antiviral responses by limiting the JAK-STAT signaling (23). ZIKV NS5 suppresses IFN-β production by targeting IRF3 and TBK1 (24, 25). Here, we reveal that ZIKV NS5 antagonizes IFN-β production by targeting RIG-I. NS5 interacts with RIG-I to inhibit RIG-I K63-linked polyubiquitination, IRF3 phosphorylation and nuclear translocation, and IFN-β production, thereby repressing the RIG-I signaling. More interestingly, NS5 conservative site D146 is required for NS5 in the suppression of RIG-I. Thus, this work uncovers an essential function of conservative site D146 in the regulation of IFN-β production and RIG-I signaling, and reveals a distinct mechanism by which ZIKV restricts antiviral responses.

## Materials and Methods

### Cell Lines and Cultures

Vero (ATCC, #CCL-81), C6/36 (ATCC, #CRL-1660), HeLa (ATCC, #CCL-2), A549 (ATCC, #CCL-185), and HEK293T cells were purchased from the American Type Culture Collection (ATCC, Manassas, VA, USA). Vero, HeLa, A549, and HEK293T cells were cultured in Dulbecco's modified Eagle's medium (DMEM) (Gibco, Grand Island, NY, USA) supplemented with 10% FBS, 100 U/ml penicillin, and 100 μg/ml streptomycin sulfate. Vero, HeLa, A549, MEF, and HEK293T cells were maintained at 37°C in 5% CO_2_ incubator. C6/36 cells were maintained in an incubator at 30°C with 5% CO_2_. IFNAR1^−/−^ MEF cells were derived from 129/Sv/Ev mice (A129 mice). The free of mycoplasma contamination of Vero and C6/36 cell stocks used in this study was tested by using the MycoTest Kit (ChanGEnome, China) and by using transmission electron microscope (TEM).

### Reagents

DMEM were purchased from Gibco (Grand Island, NY, USA). Antibody against Flag (F3165 or F1804) (1:2000), antibody against HA (1:1000), and monoclonal mouse anti-GAPDH (G9295) (1:5000) were purchased from Sigma (St. Louis, MO, USA). Antibody against GFP (catalog no. 66002-1-Ig) (1:1000) was purchased from Proteintech Group (Chicago, IL, USA). Antibody against p-IRF3 at Ser396 (4947s) (1:1000) was purchased from Cell Signaling Technology (CST, Boston, MA, USA). Antibody against IRF3 (sc-9082) (1:1000) was purchased from Santa Cruz Biotechnology (Dallas, TX, USA). Antibody against RIG-I(D1466) (1:1000) was purchased from Cell Signaling Technology (Beverly, MA, USA). Antibody against Mono-Methyl Lysine (14679S) (1:1000) was purchased from Cell Signaling Technology. S-(5′-Adenosyl)-L-homocysteine (SAH) was purchased from Sigma. Polyinosinic–polycytidylic acid poly(I:C) was purchased from InvivoGen (San Diego, CA, USA). Lipofectamine2000, normal rabbit immunoglobulin G (IgG), and normal mouse IgG were purchased from Invitrogen Corporation (Carlsbad, CA, USA).

### Viruses

The ZIKV isolate z16006 (GenBank accession number, KU955589.1) isolated by the Institute of Pathogenic Microbiology, Center for Disease Control and Prevention of Guangdong (Guangzhou, Guangdong, China) was used in this study. C6/36 cells were maintained at 30°C in DMEM (Gibco) (Grand Island, NY, USA) supplemented with 10% heat-inactivated FBS with penicillin and streptomycin (Gibco) (Grand Island, NY, USA) and 1% tryptose phosphate broth (Sigma) (St. Louis, MO, USA). To free the ZIKV stocks used in this study of mycoplasma contamination, it was tested by using the MycoTest Kit (ChanGEnome, Chian) and by using TEM. SeV was propagated in embrocated eggs and titrated by blood coagulation test.

### Plasmids Construction

IFN-β-Luc reporter plasmids were gifted from Dr. Ying Zhu of Wuhan University, China. pHA-UB, pHA-K63, pHA-K48, pHA-K63R, and pHA-K48R plasmids were gifted from Dr. Bo Zhong of Wuhan University, China. Mammalian expression plasmids for HA-, Flag-, or GFP-tagged RIG-I or Flag-tagged MAVS, TBK1, IKKε, IRF3, and IRF3/5D were constructed by standard molecular cloning method from cDNA templates. The expression plasmids CARD domain (1–256 aa) of RIG-I protein were cloned into pCAGGS-HA vector or pEGFP-C1 vector. The expression plasmids DExD/H-box domain (257–735 aa) and repressor domain (RD) (736–925 aa) of RIG-I protein were cloned into pCAGGS-HA vector. The expression plasmids ZIKV NS5 proteins were cloned into pcDNA3.1 (+)-3 × Flag vector and pCAGGS-HA vector corresponding fragments of ZIKV cDNA. The expression plasmids MTase domain, RDRP domain, K61A mutant, D146A mutant, K182A mutant, and E218A mutant of ZIKV NS5 were cloned into pcDNA3.1 (+)-3 × Flag vector or pCAGGS-HA vector. The PCR primers used in this study are summarized in Table 1.

**Table 1:** The PCR primers used in this study.

**Plasmids and genes**	**Sense primers**	**Anti-sense primers**
pcDNA3.1(+)-3Flag-RIG-I	5′-CGGGGTACCATGACCACCGAGCAGCGACGCAGCCT-3′	5′-CGGGGTACCTCATTTGGACATTTCTGCTGGATCAA-3′
pcDNA3.1(+)-3Flag-RIG-I(2CARD)	5′-CGGGGTACCATGACCACCGAGCAGCGACGCAGCCT-3′	5′-CCGCTCGAGTCATTTCATAGCAGGCAAAGCAAGCT-3′
pcDNA3.1(+)-3Flag-NS5	5″-CGGGGTACCATGGGGGGTGGAACAGGAGAGACCCT-3′	5′-CGGGGTACCATGACCACCGAGCAGCGACGCAGCCT-3′
pcDNA3.1(+)-3Flag-NS5-K61A mutant	5′-GCTGTGTCCCGAGGAAGTGCAGCGCTGAGATGGTTGGTGGAGCGGGG-3′	5′-ATCCCCGCTCCACCAACCATCTCAGCGCTGCACTTCCTCGGGACACAG-3′
pcDNA3.1(+)-3Flag-NS5-D146A mutant	5′′-GAGCCGTGTGACACGTTGCTGTGTGCCATAGGTGAGTCATCATCTAGT-3′	5′-TTCAGGACTAGATGATGACTCACCTATGGCACACAGCAACGTGTCACA-3′
pcDNA3.1(+)-3Flag-NS5-K182A mutant	5′-AAAAGACCAGGAGCCTTTTGTATAGCAGTGTTGTGCCCATACACCAG-3′′	5′GTGTATGGGCACAACACTGCTATACAAAAGGCTCCTGGTCTTTTTTC3′
pcDNA3.1(+)-3Flag-NS5-E218A mutant	5′-CTCTCCCGCAACTCTACACATGCGATGTACTGGGTCTCTGGAGCGAAA-3′	5′-TCGCTCCAGAGACCCAGTACATCGCATGTGTAGAGTTGCGGGAGAGTG-3′
pcDNA3.1(+)-3Flag-NS5-MT domain	5′-CGGGGTACCATGGGGGGTGGAACAGGAGAGACCCT-3′	5′-CCGGAATTCTTAGTTGGGAGCTTCAGCGCAGCTTA-3′
pcDNA3.1(+)-3Flag-NS5-RDRP domain	5′-CGGGGTACCATGAAGATCATTGGTAACCGCATTGA-3′	5′-CCGGAATTCTTACAGCACTCCAGGTGTAGACCCTT-3′
pcDNA3.1(+)-3Flag-STAT1	5′-CGGGGTACCTCTCAGTGGTACGAACTTCAGCAGCT-3′	5′-CCGGAATTCTTACACTTCAGACACAGAAATCAACT-3′
pCAGGS-HA-NS5-D146A mutant	5′-CGGGGTACCATGGGGGGTGGAACAGGAGAGACCCT-3′	5′-CCGCTCGAGCAGCACTCCAGGTGTAGACCCTTCTT-3′
pCAGGS -HA-NS5	5′′-CGGGGTACCATGGGGGGTGGAACAGGAGAGACCCT-3′	5′-CCGCTCGAGCAGCACTCCAGGTGTAGACCCTTCTT-3′
pCAGGS -HA-RIG-I	5′-CGGGGTACCATGACCACCGAGCAGCGACGCAGCCT-3′	5′-CCGCTCGAGTTCATTTGGACATTTCTGCTGGATCA-3′
pCAGGS-HA-RIG-I(2CARD)	5′-CGGGGTACCATGACCACCGAGCAGCGACGCAGCCT-3′	5′-CCGCTCGAGTTTCATAGCAGGCAAAGCAAGCTCTA-3′
pCAGGS-HA-RIG-I-DExD/H	5′-CGGGGTACCATGGGAAAAAACACAATAATATGTGC-3′	5′-CCGCTCGAGTCTTGCTCTTCCTCTGCCTCTGGTTT-3′
pCAGGS -HA-RIG-I-RD	5′-CGGGGTACCATGGGTAGCAAGTGCTTCCTTCTGAC-3′	5′-CCGCTCGAGTTCATTTGGACATTTCTGCTGGATCA-3′
pEGFP-C1-RIG-I	5′-CCGCTCGAGTTATGACCACCGAGCAGCGACGCAGC-3′	5′-CCCAAGCTTTCATTTGGACATTTCTGCTGGATCAA-3′
pEGFP-C1-RIG-I(2CARD)	5′-CCGCTCGAGTTATGACCACCGAGCAGCGACGCAGC-3′	5′-CCCAAGCTTTCATTTCATAGCAGGCAAAGCAAGCT-3′

### Quantitative Reverse Transcription-PCR (qRT-PCR)

Total intracellular RNA was extracted with TRIZOL reagent (Invitrogen) according the manufacturer's instructions. Real-time quantitative reverse transcriptase PCR was performed using the Roche LC480 and SYBR RT-PCR Kits (DBI Bio-science, Ludwigshafen, Germany). The reaction mixture contains 10 μl of SYBR Green PCR master mix, 1 μl of DNA diluted template, 1 μl of primers and 8 μl of RNase-free water. The following primers were used. For human IFN-β: Sense primer, 5′-AGGACAGGATGAACTTTGAC-3′; Anti-sense primer, 5′-TGATAGACATTAGCCAGGAG-3′. For human GAPDH: Sense, 5′-ATGACATCAAGAAGGTGGTG-3′; Anti-sense, 5′-CATACCAGGAAATGAGCTTG-3′.

### Luciferase Reporter Assays

In a 24-well plate, 60%−70% confluent HEK293T cells were transfected with a mixture of 200 ng of luciferase reporter (firefly luciferase) and 20 ng of pRL-TK (Renilla luciferase plasmid), together with an indicated variety expression plasmid or empty vector plasmid. HEK293T cells were co-transfected with RIG-I(2CARD), MAVS, TBK1, IKKε, or IRF3(5D) for 24 h and then stimulated with poly(I:C) for 16 h or infected with SeV for 16 h. Luciferase activity was measured by using a Dual-Luciferase Reporter Assay System kit (Promega, San Luis Obispo, CA) according to the manufacturer's protocol. Data represent relative firefly luciferase activity, normalized to Renilla luciferase activity.

### Western Blotting and Co-immunoprecipitation Assay

Cells lysates were from lysing cells with lysis buffer (50 mM Tris–HCl, pH 7.5, 300 mM NaCl, 1% Triton-X, 5 mM EDTA, and 10% glycerol). Cell lysates were immunoprecipitated with control mouse immunoglobulin G (IgG) (Invitrogen) or anti-Flag antibody (Sigma, F3165) with Protein-G Sepharose (GE Healthcare, Milwaukee, WI, USA). Protein concentration was determined by Bradford assay (Bio-Rad, Hercules, CA, USA). Cultured cell lysates (30 μg) were electrophoresed in an 8–10% SDS-PAGE gel and transferred to a PVDF membrane (Millipore, MA, US). PVDF membranes were blocked with 5% skim milk in phosphate-buffered saline (PBS) with 0.1% Tween 20 (PBST) before being incubated with the antibody. Protein band were detected using a Luminescent image Analyzer (Fujifilm LAS-4000).

### Nuclear and Cytoplasmic Extraction

In a 12-well plate, 60–70% confluent HeLa cells were transfected with the indicated plasmids for 24 h, then disposed by using by nuclear and cytoplasmic extraction reagents (Thermo scientific, 78833, USA). Cytosol or nuclear lysate concentration was determined by Bradford assay (Bio-Rad, Hercules, CA, USA).

### Confocal Microscopy

In a 24-well plate, 40%−50% HEK293T or HeLa cells were transfected with the indicated plasmids (500 ng) for 24 h, then cells were washed twice with PBS and fixed in 4% paraformaldehyde at room temperature for 10 min, then permeabilized with wash buffer (PBS containing 0.1% Triton X-100) for 5 min, washed three times with PBS, and finally blocked with PBS containing 5% BSA for 1 h. The cells were then incubated with the primary antibody overnight at 4°C, followed by incubated with FITC-conjugate donkey anti-mouse IgG and Dylight 649-conjugate donkey anti-rabbit IgG (Abbkine) for 1 h. Using wash buffer three times, cells were incubated with DAPI solution for 5 min, and then washed three more times with PBS. Finally, the cells were analyzed using a confocal laser scanning microscope (Fluo View FV1000; Olympus, Tokyo, Japan).

### Statistical Analyses

All experiments were reproducible and repeated at least three times with similar results. Samples were analyzed by one-way analysis of variance with Tukey's *post-hoc* test. Abnormal values were eliminated using a follow-up Grubbs test. A Levene's test for equality of variances was performed, which provided information for Student's *t*-tests to distinguish the equality of means. Means were illustrated using histograms, with error bars representing standard error of the mean (s.e.m); values of *P* < 0.05 were considered to indicate statistical significance (^*^P < 0.05, ^**^P < 0.01, and ^***^P < 0.001).

## Results

### ZIKV NS5 Represses IFN-β Production by Targeting the RIG-I Pathway

**Figure 1:**
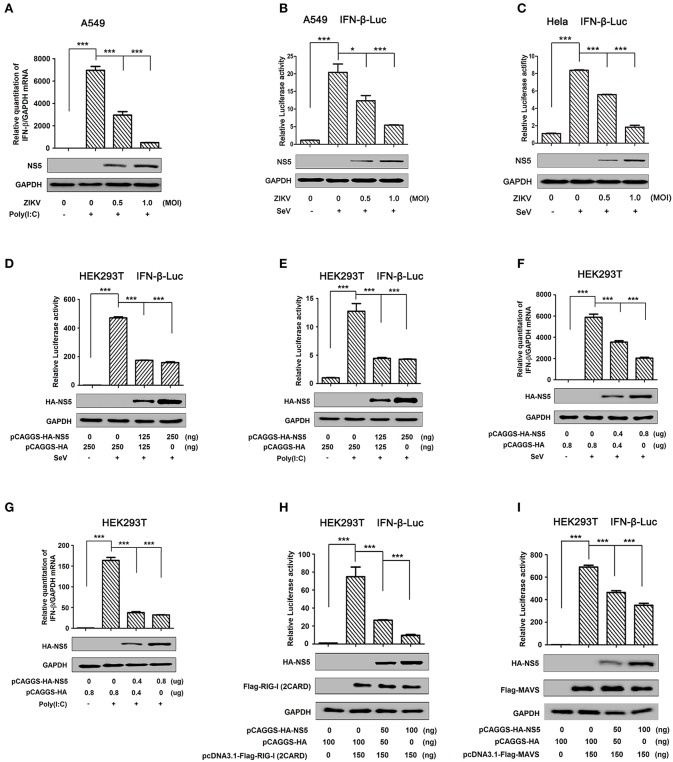
ZIKV NS5 represses IFN-β production by targeting the RIG-I pathway. **(A)** A549 cells were infected with ZIKV (0.5 or 1 MOI) and transfected with cytoplasmic poly(I:C) (5 μg/ml). IFN-β mRNA was determined by quantitative PCR (top). The infection of ZIKV was confirmed by Western blotting analysis of NS5 (bottom). **(B,C)** A549 cells **(B)** or Hela cells **(C)** were transfected with IFN-β luciferase reporter pIFN-β-Luc and pPRL-TK for 24 h, then infected with ZIKV (0.5 or 1 MOI), and then stimulated with Sendai virus (SeV) (0.1 MOI) for 12 h. Cell lysates were harvested, IFN-β-Luc reporter activity was determined by dual luciferase reporter assays (top), and HA-NS5 was detected by Western blotting (bottom). **(D,E)** HEK293T cells were co-transfected with IFN-β luciferase reporter pIFN-β-Luc and pPRL-TK together with pHA-NS5 for 24 h and then infected with Sendai virus (SeV) (0.1 MOI) for 16 h **(D)** or transfected with cytoplasmic poly(I:C) (2 μg/ml) for 16 h **(E)**. Cell lysates were harvested, IFN-β-Luc reporter activity was determined by dual luciferase reporter assays (top), and HA-NS5 was detected by Western blotting (bottom). **(F,G)** HEK293T cells were transfected with pHA-NS5 for 24 h and infected with SeV (MOI = 0.1) for 16 h **(F)** or transfected with poly(I:C) (2 μg/ml) for 16 h **(G)**. IFN-β mRNA was determined by q-PCR (top) and HA-NS5 was confirmed by Western blotting (bottom). **(H,I)** HEK293T cells were co-transfected with pIFN-β-Luc, pPRL-TK, and pHA-NS5, together with pFlag-RIG-I-(2CARD) **(H)** and pFlag-MAVS **(I)** for 24 h. Cell lysates were harvested, IFN-β-Luc reporter activity was determined by dual luciferase reporter assays (top), and HA-NS5, Flag-RIG-I-(2CARD), and Flag-MAVS were confirmed by Western blotting (bottom). Data in A–I were expressed as means ± s.e.m. of at least three independent experiments. **P < 0.01, ***P < 0.001.

IFN-β plays an important role in activating immune cells and suppressing virus replication (26–31), and ZIKV infection leads to low levels of type I IFNs (32). Here, we initially showed that IFN-β mRNA was significantly induced by poly(I:C), but such induction was suppressed by ZIKV infection (Figure 1A). Additionally, IFN-β-Luc activity was induced upon Sendai virus (SeV) infection, but the induction was suppressed by ZIKV in A549 cells (Figure 1B) or Hela cells (Figure 1C). These results demonstrate that ZIKV suppresses IFN-β expression by the stimulation of poly(I:C) or SeV. IFN-β-Luc activity was induced upon Sendai virus (SeV) infection (Figure 1D) or by poly(I:C) treatment (Figure 1E), but the induction was suppressed by NS5 in HEK293T cells (Figures 1D,E). Moreover, endogenous IFN-β mRNA was induced upon SeV infection (Figure 1F) and by poly(I:C) treatment (Figure 1G), but such induction was attenuated by NS5 (Figures 1F,G). These results demonstrate that NS5 suppresses IFN-β expression upon the infections of SeV or by the stimulation of poly(I:C). Since ZIKV genome is recognized by RIG-I, we investigated whether NS5 affects RIG-I function. Overexpression of NS5 in HEK293 cells attenuated the activation of IFN-β promoter luciferase reporter activity by RIG-I and MAVS (Figures 1H,I). Taken together, we demonstrate that ZIKV suppresses IFN-β production by repressing the RIG-I signaling through NS5.

#### NS5 Restricts IRF3 Phosphorylation and Nuclear Translocation

**Figure 2:**
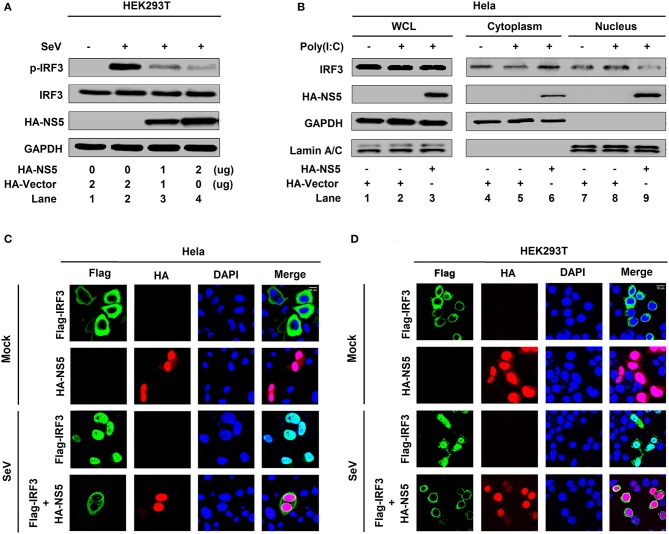
NS5 restricts IRF3 phosphorylation and nuclear translocation. **(A)** HEK293T cells were transfected with pHA-NS5 for 24 h and infected with SeV (MOI = 0.1) for 16 h. Phosphorylated IRF3, total IRF3, NS5, and GAPDH were subjected to Western blotting with indicated antibodies anti-pIRF3, anti-IRF3, anti-HA, and anti-GAPDH, respectively. **(B)** Hela cells were transfected with pHA-NS5 for 24 h and treated with poly(I:C) (5 μg/ml) for 16 h. IRF3 in the nuclear fractions or the cytoplasmic was determined by immunoblotting analyses. GAPDH was served as a cytoplasmic control and Lamin A/C was served as a nuclear protein control. **(C,D)** Hela cells **(C)** or HEK293T cells **(D)** were transfected with pFlag-IRF3 or pHA-NS5 or co-transfected with pFlag-IRF3 and pHA-NS5 and infected with SeV (MOI = 0.1) for 16 h. The sub-cellular localizations of Flag-IRF3 (green), HA-NS5 (red), and nucleus marker DAPI (blue) were analyzed with confocal microscopy.

IRF3 is an important component of the RIG-I pathway and activation of IRF3 depends on phosphorylation, which leads to IRF3 nuclear localization and IFN-β production (18). Here, we investigated the effect of NS5 on IRF3 phosphorylation and nuclear translocation. IRF3 phosphorylation was induced upon SeV infection (Figure 2A, lane 2 vs. 1), whereas NS5 significantly repressed SeV-induced IRF3 phosphorylation but not IRF3 production (Figure 2A, lanes 3 and 4 vs. 2). Additionally, in the cytoplasmic extraction of Hela cells, IRF3 was down-regulated by poly(I:C) (Figure 2B, lane 5 vs. 4) and enhanced by NS5 (Figure 2B, lane 6 vs. 5); however, in the nuclear extraction of Hela cells, IRF3 was up-regulated by poly(I:C) (Figure 2B, lane 8 vs. 7) but attenuated by NS5 (Figure 2B, lane 9 vs. 8). Moreover, in mock-infected HEK293T cells (Figure 2C) and Hela cells (Figure 2D), IRF3 alone was mainly distributed in the cytoplasm, a small proportion of NS5 was located in the cytoplasm, and a large proportion of NS5 was distributed in the nucleus; however, in SeV-infected HEK293T cells (Figure 2C) and Hela cells (Figure 2D), IRF3 was translocated from the cytoplasm to the nucleus in the absence of NS5; and interestingly, most of IRF3 remained in the cytoplasm in the presence of NS5. Taken together, the results reveal that NS5 restricts IRF3 phosphorylation and nuclear translocation, but not IRF3 production, thereby repressing IRF3 activation.

### NS5 Binds to the CARD Domain of RIG-I

**Figure 3:**
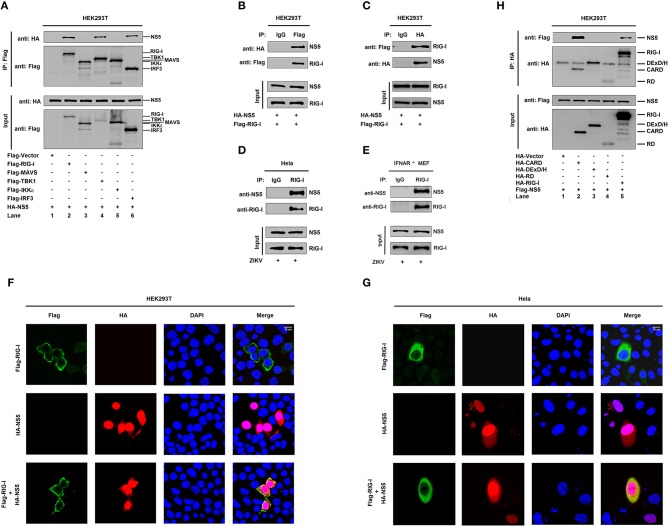
NS5 binds to the CARD domain of RIG-I. **(A)** HEK293T cells were co-transfected with pHA-NS5 in combination with pFlag-Vector, pFlag-RIG-I, pFlag-MAVS, pFlag-TBK1, pFlag-IKKε, or pFlag-IRF3. Cell lysates were subjected to immunoprecipitation (IP) using anti-Flag antibody and analyzed by immunoblotting using anti-HA and anti-Flag antibody (top). Cell lysates (30 μg protein) were analyzed directly by immunoblotting using anti-HA and anti-Flag antibody as input (bottom). **(B,C)** HEK293T cells were co-transfected with pHA-NS5 and pFlag-RIG-I. Cell lysates were subjected to IP using control IgG or anti-Flag antibody **(B)** and control IgG or anti-HA antibody **(C)**. Cell lysates (30 μg protein) were analyzed directly by immunoblotting using anti-HA and anti-Flag antibody as input (bottom) **(B,C)**. **(D)** Hela cells were infected with ZIKV (MOI = 1) for 24 h. Cell lysates were subjected to IP using control IgG and anti-RIG-I antibody and then analyzed by immunoblotting using anti-NS5 antibody and anti-RIG-I antibody. Cell lysates (30 μg protein) were analyzed by immunoblotting using anti-NS5 and anti-RIG-I antibody as input (bottom). **(E)** IFNAR^−/−^ MEF cells were infected with ZIKV (MOI = 1) for 24 h. Cell lysates were subjected to IP using control IgG and anti-RIG-I antibody and then analyzed by immunoblotting using anti-NS5 antibody and anti-RIG-I antibody. Cell lysates (30 μg protein) were analyzed by immunoblotting using anti-NS5 and anti-RIG-I antibody as input (bottom). **(F,G)** HEK293T cells **(F)** and Hela cells **(G)** were transfected with pFlag-RIG-I or pHA-NS5, or co-transfected with pFlag-RIG-I and pHA-NS5. The sub-cellular localizations of Flag-RIG-I (green), HA-NS5 (red), and nucleus marker DAPI (blue) were analyzed with confocal microscopy. **(H)** HEK293T cells were co-transfected with pHA-NS5 in combination with pFlag-Vector, pFlag-RIG-I-(2CARD), pFlag-DExD/H, or pFlag-RD. Cell lysates were subjected to IP using anti-Flag antibody and then analyzed by immunoblotting using anti-HA and anti-Flag antibody (top). Cell lysates (30 μg protein) were analyzed directly by immunoblotting using anti-HA and anti-Flag antibody as input (bottom).

The mechanism by which NS5 represses the RIG-I signaling was elucidated. Initially, we determined whether NS5 interacts with the RIG-I signaling components. Interestingly, NS5 interacted with RIG-I, TBK1, and IRF3 (Figure 3A, lanes 2, 4, and 6), but failed to interact with MAVS or IKKε (Figure 3A, lanes 3 and 5). Co-IP further confirmed that NS5 associated with RIG-I (Figure 3B) and RIG-I interacted with NS5 (Figure 3C). Additionally, NS5 was co-immunoprecipitated with endogenous RIG-I in ZIKV-infected Hela cells and IFNAR^−/−^ MEF cells (Figures 3D,E). Moreover, in HEK293T cells (Figure 3F) and Hela cells (Figure 3G), RIG-I was diffusely distributed in the cytoplasm, a small proportion of NS5 was distributed in the cytoplasm, and a large proportion of NS5 was distributed in the nucleus; however, most of RIG-I was internalized in the cytoplasm and a proportion of NS5 was co-localized with RIG-I in the cytoplasm (Figures 3F,G). Thus, these results demonstrate that NS5 interacts with RIG-I. Since RIG-I contains three domains, CARD, DExD/H, and RD (14), we determined which domain is involved in the interaction with NS5 and revealed that NS5 interacted with RIG-I and CARD domain (Figure 3H, lanes 2 and 5), but not with DExD/H domain or RD domain (Figure 3H, lanes 3 and 4). Therefore, the results demonstrate that NS5 binds to the CARD domain of RIG-I.

### NS5 Attenuates K63-Linked Polyubiquitination of RIG-I

**Figure 4:**
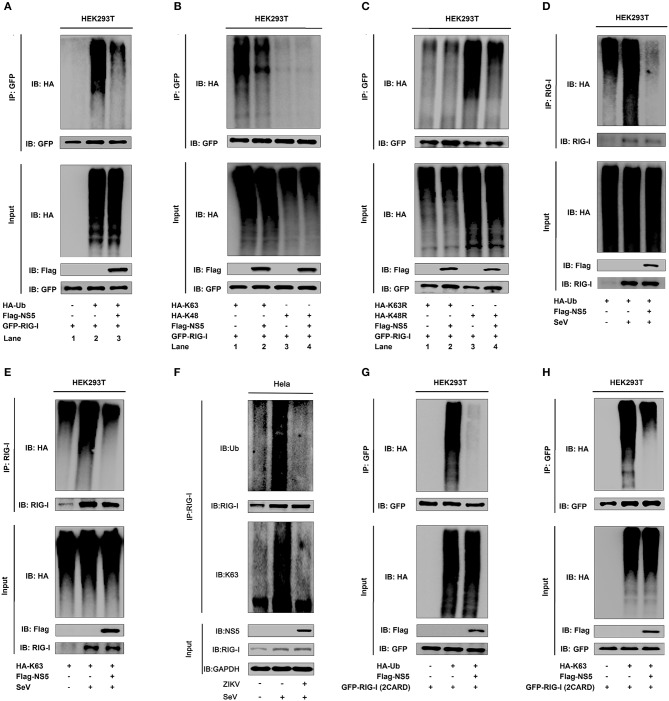
NS5 attenuates K63-linked polyubiquitination of RIG-I. **(A)** HEK293T cells were co-transfected with pGFP-RIG-I, pHA-Ub, and pFlag-NS5. Cell lysates were immunoprecipitated with anti-GFP and immunoblotted with anti-HA. **(B)** HEK293T cells were co-transfected with pGFP-RIG-I, pHA-K63, or pHA-K48, together with pFlag-NS5. Cell lysates were immunoprecipitated with anti-GFP and immunoblotted with anti-HA. **(C)** HEK293T cells were co-transfected with pGFP-RIG-I and pHA-K63R or pHA-K48R, together with pFlag-NS5. Cell lysates were immunoprecipitated with anti-GFP and immunoblotted with anti-HA. **(D)** HEK293T cells were co-transfected with pHA-Ub and pFlag-NS5 and infected with SeV (MOI = 0.1) for 16 h. Cell lysates were subjected to IP with anti-RIG-I and immunoblot analysis with anti-HA. **(E)** HEK293T cells were co-transfected with pHA-K63, together with pFlag-NS5 and infected with SeV (MOI = 0.1) for 16 h. Cell lysates were subjected to IP with anti-RIG-I and immunoblot analysis with anti-HA. **(F)** Hela cells were infected with ZIKV (MOI = 1) for 12 h and stimulated with SeV (MOI = 0.1) for 6 h. Cell lysates were subjected to IP with anti-RIG-I and immunoblot analysis with anti-Ub or anti-K63. **(G)** HEK293T cells were co-transfected pGFP-RIG-I-(2CARD) and pHA-Ub, together with pFlag-NS5. Cell lysates were immunoprecipitated with anti-GFP and immunoblotted with anti-HA. **(H)** HEK293T cells were co-transfected with pGFP-RIG-I-(2CARD) and pHA-K63, together with pFlag-NS5. Cell lysates of were immunoprecipitated with anti-GFP and immunoblotted with anti-HA.

Ubiquitination plays critical roles in the regulation of immune signaling pathways (33). The ubiquitination of RIG-I mainly occurs on its CARD domain and NS5 binds to the CARD domain of RIG-I (34), and we found that NS5 binds to the CARD domain of RIG-I. Here, the role of NS5 in the regulation of RIG-I polyubiquitination was determined. RIG-I polyubiquitination was catalyzed by HA-Ub (Figure 4A, lanes 1 and 2), but reduced by NS5 (Figure 4A, lane 3 vs. 2). Additionally, RIG-I polyubiquitination was catalyzed by HA-K63 (ubiquitin mutant retaining a single lysine at K63) (Figure 4B, lanes 1 and 2), and K63-liked polyubiquitination was attenuated by NS5 (Figure 4B, lane 2 vs. 1), while RIG-I polyubiquitination was weakly catalyzed by HA-K48 (ubiquitin mutant retaining a single lysine at K48) (Figure 4B, lane 3), and RIG-I K48-liked polyubiquitination was not affected by NS5 (Figure 4B, lane 4). Moreover, RIG-I polyubiquitination was weakly catalyzed by K63R (lysine 63 of ubiquitin was mutated into arginine) (Figure 4C, lane 1), which was not affected by NS5 (Figure 4C, lane 2); however, RIG-I polyubiquitination was strongly catalyzed by K48R (lysine 48 of ubiquitin was mutated into arginine) (Figure 4C, lane 3), which was attenuated by NS5 (Figure 4C, lane 4). The effects of NS5 on the regulation of RIG-I polyubiquitination upon virus infection or RIG-I signaling activation was then investigated. RIG-I polyubiquitination catalyzed by HA-Ub (Figure 4D) or HA-K63 (Figure 4E) was induced upon SeV infection, but such induction was repressed by NS5 (Figures 4D,E). Interestingly, RIG-I polyubiquitination catalyzed by Ub (Figure 4F, upper) or K63 (Figure 4F, lower) was induced by SeV, but such induction was repressed by ZIKV infection (Figure 4F). RIG-I possesses two caspase activation and recruitment domains (CARDs), a DExD/H-box helicase domain, and a repressor domain (RD), and RIG-I undergoes robust ubiquitination at its N-terminal CARD domain. We showed that RIG-I(2CARD) polyubiquitination was catalyzed by HA-Ub (Figure 4G) or by HA-K63 (Figure 4H), but attenuated by NS5 (Figures 4G,H). Taken together, we demonstrate that NS5 impairs K63-linked polyubiquitination of RIG-I CARD domain.

### Conservative Site D146 Is Essential for NS5 in the Suppression of IFN-β

**Figure 5:**
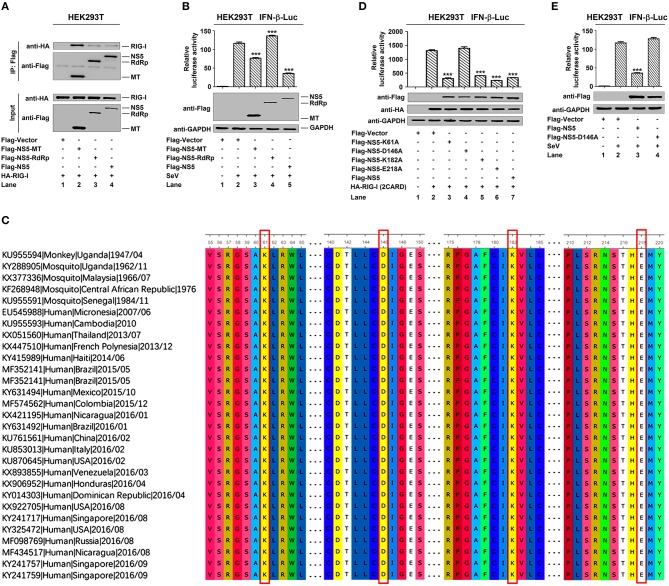
MTase active site D146 is essential for NS5 in the suppression of IFN-β. **(A)** HEK293T cells were co-transfected with pHA-RIG-I in combination with pFlag-NS5-MT, pFlag-NS5-RdRp, or pFlag-NS5. Cell lysates were subjected to IP using control IgG and anti-Flag antibody and analyzed by immunoblotting using anti-HA and anti-Flag antibody (top). Cell lysates (30 μg protein) were analyzed by immunoblotting using anti-HA and anti-Flag antibody as input (bottom). **(B)** HEK293T cells were co-transfected with pIFN-β-Luc and pPRL-TK, together with pFlag-NS5-MT, pFlag-NS5-RdRp, or pFlag-NS5 for 24 h and infected with SeV (MOI = 0.1) for 16 h. Cell lysates were harvested, IFN-β-Luc reporter activity was determined by dual luciferase reporter assays (top), and NS5-MT, NS5-RdRp, or NS5 were confirmed by Western blotting (bottom). **(C)** Sequence comparison was performed using MEGA6 software. The Genbank accession numbers and isolated years of the ZIKV strains were listed. Variations were presented in accordance with their locations in viral genome. **(D)** HEK293T cells were co-transfected pIFN-β-Luc, pPRL-TK, and pHA-RIG-I-(2CARD), together with pFlag-NS5-K61A, pFlag-NS5-D146A, pFlag-NS5-K182A, pFlag-NS5-E218A, or pFlag-NS5 for 24 h. IFN-β-Luc activity was determined by dual luciferase reporter assays (top), and HA-RIG-I(2CARD), Flag-NS5-K61A, Flag-NS5-D146A, Flag-NS5-K182A, Flag-NS5-E218A, or Flag-NS5 were confirmed by Western blotting (bottom). **(E)** HEK293T cells were co-transfected pIFN-β-Luc and pPRL-TK together with pFlag-NS5 or pFlag-NS5-D146A for 24 h and infected with SeV (MOI = 0.1) for 16 h. IFN-β-Luc reporter activity was determined by dual luciferase reporter assays (top), and NS5 and NS5-D146A were confirmed by Western blotting (bottom). Data in **(B,D,E)** were expressed as means ± s.e.m. of at least three independent experiments. **P < 0.01, ***P < 0.001.

NS5 plays a key role in the replication of viral genome and contains a methyltransferase (MTase) domain and an RNA-dependent RNA polymerase (RdRp) domain (35, 36). Here, we determined whether MTase and RdRp are involved in this regulation. RIG-I interacted strongly with NS5-MTase (Figure 5A, lane 2) and interacted weakly with NS5-RdRp or NS5 (Figure 5A, lanes 3 and 4). Additionally, IFN-β-Luc activity was induced upon SeV infection (Figure 5B, lane 2 vs. 1), but this induction was attenuated by NS5-MTase (Figure 5B, lane 3 vs. 2), repressed by NS5 (Figure 5B, lane 5 vs. 2), and facilitated by NS5-RdRp (Figure 5B, lane 4 vs. 2). Therefore, these results reveal that like NS5, MTase domain interacts with RIG-I and represses IFN-β activation.

Like all amino acids, compared to the original 1947 sequence, the conservative catalytic tetrad K61-D146-K182-E218 is positioned in the center of MTase domain to form the active site of MTase (Figure 5C) (37). We investigated the effect of conservative site on the regulation of IFN-β by generating and analyzing conservative site mutants, NS5-K61A, -D146A, -K182A, and -E218A. IFN-β-Luc activity was induced by RIG-I(2CARD) (Figure 5D, lane 2 vs. 1), and such activation was repressed by NS5-K61A (Figure 5D, lane 3 vs. 2), NS5-K182A (Figure 5D, lane 5 vs. 2), NS5-E218A (Figure 5D, lane 6 vs. 2), and NS5 (Figure 5D, lane 7 vs. 2), but not affected by NS5-D146A (Figure 5D, lane 4 vs. 2). Additionally, IFN-β-Luc activity was induced by SeV (Figure 5E, lane 2 vs. 1), and such induction was suppressed by NS5 (Figure 5E, lane 3 vs. 2) but not by NS5-D146A (Figure 5E, lane 4 vs. 2). These results reveal that the conservative site D146 is essential for NS5 in the repression of IFN-β.

### Conservative Site D146 Is Required for the Repression of RIG-I Signaling

**Figure 6:**
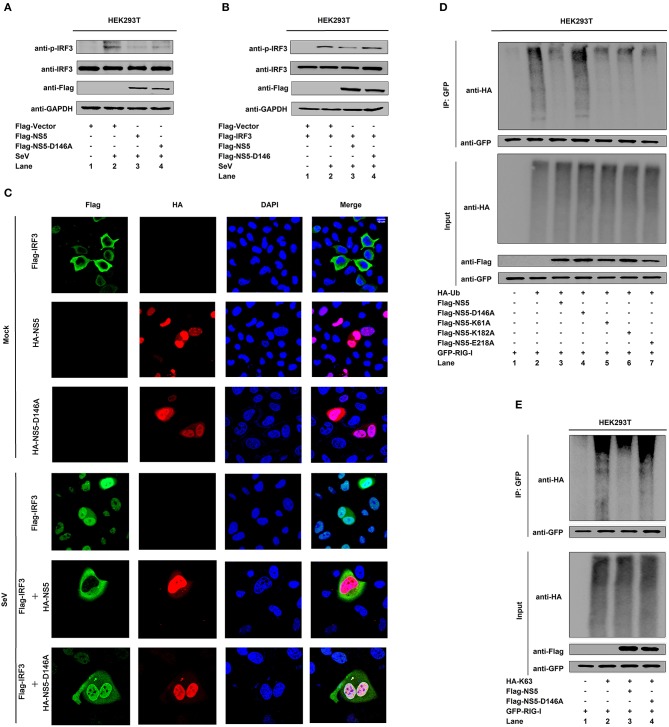
NS5 MTase active site D146 is required for the repression of RIG-I signaling. **(A,B)** HEK293T cells were transfected with pFlag-NS5 or pFlag-NS5-D146A for 24 h and infected with SeV (MOI = 0.1) for 16 h **(A)** or co-transfected with pFlag-IRF3 together with pFlag-NS5 or pFlag-NS5-D146A for 24 h and infected with SeV (MOI = 0.1) for 16 h **(B)**. The phosphorylated IRF3, total IRF3, NS5, NS5-D146A, and GAPDH proteins were detected by Western blotting with indicated antibodies, anti-pIRF3, anti-IRF3, anti-Flag, anti-Flag, and anti-GAPDH. **(C)** Hela cells were transfected with pFlag-IRF3, pHA-NS5, or pHA-NS5-D146A, and co-transfected with pFlag-IRF3 and pHA-NS5 or pFlag-IRF3 and pHA-NS5-D146A and infected with SeV (MOI = 0.1) for 16 h. The sub-cellular localizations of Flag-IRF3 (green), HA-NS5 (red), HA-NS5-D146A mutant (red), and nucleus marker DAPI (blue) were analyzed with confocal microscopy. **(D)** HEK293T cells were co-transfected with pGFP-RIG-I and pHA-Ub, together with pFlag-NS5, pFlag-NS5-D146A, pFlag-NS5-K61A, pFlag-NS5-K182A, or pFlag-NS5-E218A. Cell lysates were immunoprecipitated with anti-GFP and immunoblotted with anti-HA. **(E)** HEK293T cells were co-transfected with pGFP-RIG-I and pHA-K63, together with pFlag-NS5 or pFlag-NS5-D146A. Cell lysates were immunoprecipitated with anti-GFP and immunoblotted with anti-HA.

Since NS5 represses the RIG-I signaling, we evaluated the effect of D146 on such regulation. The phosphorylation of endogenous IRF3 was induced upon SeV infection (Figure 6A, lane 2 vs. 1), repressed by NS5 (Figure 6A, lane 3 vs. 2), and slightly down-regulated by NS5-D146A (Figure 6A, lane 4 vs. 2). Similarly, the phosphorylation of transfected IRF3 was activated upon SeV infection (Figure 6B, lane 2 vs. 1), attenuated by NS5 (Figure 6B, lane 3 vs. 2), but not by NS5-D146A (Figure 6B, lane 4 vs. 2). These results indicate that D146 is involved in the repression of virus-induced phosphorylation of IRF3. Moreover, in mock-infected cells, IRF3 was diffusely distributed in the cytoplasm, a small proportion of NS5 and NS5-D146A was located in the cytoplasm, and a large proportion of NS5 and NS5-D146A was distributed in the nucleus (Figure 6C, top); however, in SeV-infected cells, IRF3 was translocated from the cytoplasm to the nucleus in the absence of NS5 or in the presence of NS5-D146A, but remained in the cytoplasm in the presence of NS5 (Figure 6C); revealing that D146 is essential for NS5 in the repression of IRF3 nucleus translocation.

Since NS5 attenuates K63-linked polyubiquitination of RIG-I, we determined whether D146 plays a role in this regulation. RIG-I polyubiquitination was catalyzed by HA-Ub (Figure 6D, lane 2), repressed by NS5 (Figure 6D, lane 3), NS5-K61A (Figure 6D, lane 5), NS5-K182A (Figure 6D, lane 6), and NS5-E218A (Figure 6D, lane 7), but not affected by NS5-D146A (Figure 6D, lane 4), demonstrating that D146 is essential for the repression of RIG-I polyubiquitination. Additionally, RIG-I polyubiquitination was catalyzed by Ub-K63 (Figure 6E, lane 2), and repressed by NS5 (Figure 6E, lane 3) but not by NS5-D146A (Figure 6E, lane 4), confirming that D146 is essential for the repression of RIG-I K63-linked polyubiquitination. Taken together, we reveal that D146 is required for NS5 in the suppression of the RIG-I pathway.

### MTase Activity Does Not Contribute to NS5 Repressing RIG-I Signaling

**Figure 7:**
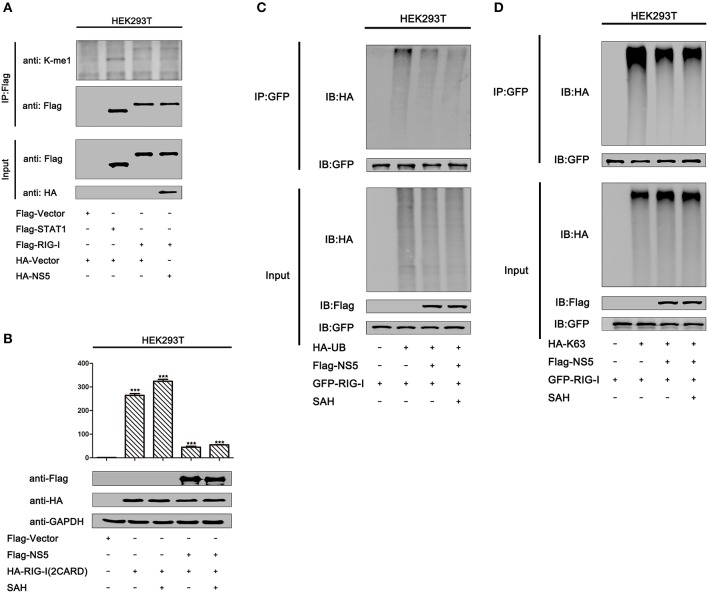
MTase activity does not contribute to NS5 repressing RIG-I signaling. **(A)** HEK293T cells were co-transfected with pFlag-vector, pFlag-STAT1, pFlag-RIG-I, and co-transfected pFlag-RIG-I and pHA-NS5. Cell lysates were subjected to IP using anti-Flag antibody, and then analyzed by immunoblotting using anti-mono-methylation-lysing antibody and anti-Flag antibody (top). Cell lysates (30 μg protein) were analyzed directly by immunoblotting using anti-anti-HA and anti-Flag antibody as input (bottom). **(B)** HEK293T cells were co-transfected with pIFN-β-Luc, pPRL-TK, and pHA-NS5, together with pFlag-RIG-I-(2CARD), then adding SAH (200 μM) for 24 h. Cell lysates were harvested, IFN-β-Luc reporter activity was determined by dual luciferase reporter assays (top), and HA-NS5 and Flag-RIG-I-(2CARD) were confirmed by Western blotting (bottom). **(C)** HEK293T cells were co-transfected with pGFP-RIG-I and pHA-Ub, together with pFlag-NS5, then adding SAH (200 μM) for 24 h. Cell lysates were immunoprecipitated with anti-GFP and immunoblotted with anti-HA. **(D)** HEK293T cells were co-transfected with pGFP-RIG-I and pHA-K63, together with pFlag-NS5, then adding SAH (200 μM) for 24 h. Cell lysates were immunoprecipitated with anti-GFP and immunoblotted with anti-HA.

Because we found that MTase site D146 is required for the repression of RIG-I signaling, we asked whether NS5 mediates methylation of RIG-I to affect its activity. We found that overexpression of the NS5 did not lead to increased mono-methylation of RIG-I in HEK293T cells (Figure 7A, lane 4 vs. 3). Moreover, we also determined whether MTase activity contributed to NS5 repressing RIG-I signaling. It has been reported that MTase inhibitors SAH can inhibit ZIKV NS5 MTase activity (38). In HEK293T cells, IFN-β-Luc activity induced by RIG-I(2CARD) was attenuated by NS5. Similarly, NS5 also attenuated the IFN-β-Luc activity in HEK293T cells adding SAH (Figure 7B). In addition, NS5 attenuates K63-linked polyubiquitination of RIG-I. We also found that adding SAH did not influence NS5 suppressing polyubiquitination or K63-linked polyubiquitination of RIG-I (Figures 7C,D). These data suggest that NS5 did not increase mono-methylation of RIG-I and its MTase activity did not contribute to NS5 repressing RIG-I signaling.

## Discussion

Host cells have developed innate immune systems to inhibit the virus invasion and replication (39), while viruses must employ strategies to evade host immune systems to maintain infection and replication (40). We have recently demonstrated that ZIKV NS5 induces host inflammatory responses by facilitating the NLRP3 inflammasome assembly and interleukin-1β secretion (41). Here, we show a distinct mechanism by which ZIKV restricts host antiviral immune responses by targeting the RIG-I signaling. ZIKV suppresses IFN-β expression mediated by SeV infection and poly(I:C) stimulation, which is consistent with a previous study showing that ZIKV infection leads to low levels of type I IFN production (32). NS5 represses IFN-β activation induced by SeV infection and poly(I:C) stimulation, suggesting that ZIKV represses type I IFN through NS5. Previous studies reported that ZIKV NS5 interferes with type I IFN signaling by targeting STAT2 for proteasomal degradation (22), while NS1 and NS4B prevent RLR pathway activation by targeting TBK1 to inhibit IFN-β, NS2B3 impairs the JAK-STAT pathway through degrading JAK1 (23) and attenuates the cGAS/STING pathway by cleaving STING (42), and ZIKV suppresses IFN-β expression mediated by SeV infection and poly(I:C) stimulation (32). Two recent studies demonstrated that NS5 limits IFN-β by interacting with IRF3 and TBKI (24, 25). Therefore, our results are consistent with the conclusions of previous reports, and further illustrate a distinct mechanism that NS5 antagonizes IFN-β production by interacting with RIG-I. IFN-β plays important roles in the activation of immune cells and the suppression of virus replication (26–31). During this process, IRF3 phosphorylation and nuclear translocation are essential for IFN-β production upon virus infection (18). We reveal that NS5 restricts IRF3 phosphorylation and nuclear translocation, thereby repressing IRF3 activation and IFN-β production.

The molecular mechanism by which NS5 suppresses IFN-β production and RIG-I signaling is revealed. Two studies have shown that NS5 blocks dsRNA-stimulated IFN response by interacting with IRF3 or TBK1 (24, 25). Interestingly, our results are consistent with their data, and we also reveal that NS5 interacts with RIG-I, TBK1, and IRF3, and fails to interact with MAVS or IKKε. Here we mainly focus on the study of the molecular mechanism by which NS5 suppresses IFN-β production by interacting with RIG-I. We further reveal that NS5 binds to RIG-I through the CARD domain. RIG-I K63-linked polyubiquitination is crucial for the pathway to elicit host antiviral immune responses. West Nile virus (WNV) and influenza A virus (IAV) have developed diverse strategies to minimize IFN by decreasing RIG-I K63-linked polyubiquitination (43, 44). Interestingly, we reveal that ZIKV represses RIG-I K63-linked polyubiquitination through NS5. These results suggest that NS5 may play a role in the regulation of viral infection through repressing RIG-I ubiquitination, since RIG-I ubiquitination plays a key role in the regulation of viral infection (34).

Functional analyses of the domains of ZIKV NS5 reveal that the MTase domain, but not the RdRp domain, represses IFN-β activation by targeting RIG-I. The conservative catalytic tetrad of K61-D146-K182-E218 is positioned in the MTase domain to form the active site of MTase (45). Our results demonstrate that the conservative site D146 is essential for NS5 in repressing IFN-β production, IRF3 activation, and RIG-I K63-linked polyubiquitination. Since D146 is an NS5 MTase catalytic site, we supposed that MTase activity may contribute to NS5 suppressing RIG-I signaling. But we found that ZIKV NS5 does not lead to increased methylation of RIG-I and NS5 MTase activity does not contribute to NS5-attenuated IFN-β activation and RIG-I K63-linked polyubiquitination. Therefore, we suggest that the conservative site D146 is important for NS5 repressing IFN-β production, IRF3 activation, and RIG-I K63-linked polyubiquitination, but it has no relation with the NS5 MTase activation. The conservative site D146 may play an important role in maintaining the NS5 space structure; this hypothesis needs to be further investigated.

In conclusion, this study reveals a distinct mechanism by which ZIKV restricts antiviral response by targeting RIG-I. NS5 binds to RIG-I through interacting with the CARD domain, resulting in the restriction of RIG-I polyubiquitination, IRF3 activation, and IFN-β production, thereby inhibiting the RIG-I signaling. These results provide evidence that ZIKV NS5 interferes with the host immune system by targeting RIG-I. More interestingly, MTase active site D146 is essential for the repression of RIG-I signaling, but MTase activity does not contribute to NS5 suppressing RIG-I signaling.

## Data Availability Statement

The raw data supporting the conclusions of this article will be made available by the authors, without undue reservation, to any qualified researcher.

## Author Contributions

AL, WW, YW, YL, KW, and JW contributed to the design of experiments. AL, WW, YW, KC, FX, DH, LH, WL, and YF contributed to the conduction of experiments. AL, WW, YW, KC, FX, DH, LH, WL, YF, GL, and QT contributed to the reagents. AL, WW, YW, KC, FX, QT, YL, KW, and JW contributed to the analyses of the data. AL, WW, KW, and JW contributed to writing the paper. AL and JW contributed to the editing of the paper.

### Conflict of Interest

The authors declare that the research was conducted in the absence of any commercial or financial relationships that could be construed as a potential conflict of interest.
